# Vascular endothelial integration of multiple biophysical stimuli

**DOI:** 10.3389/fcvm.2026.1866905

**Published:** 2026-06-17

**Authors:** Louison Blivet-Bailly, Claire Leclech, Abdul I. Barakat

**Affiliations:** LadHyX, CNRS—École Polytechnique, Institut Polytechnique de Paris, Palaiseau, France

**Keywords:** biophysical stimulus integration, endothelial cells, intraluminal pressure, mechanobiology, shear stress, stiffness, substrate topography, tensile stretch

## Abstract

By virtue of their anatomical position at the interface between the bloodstream and the blood vessel wall, vascular endothelial cells (ECs) *in vivo* are constantly subjected to a complex and highly dynamic combination of biophysical stimuli. These stimuli, which include fluid shear stress, pressure, stretch forces, curvature effects, and contact stresses due to substrate topography and rigidity, fundamentally shape EC structure and function. Numerous studies have focused on the response of ECs to single stimuli *in vitro*. For instance, EC responses to flow have been investigated since the 1980s. More recently, the impact of the physical properties of the subendothelial basement membrane including topography and rigidity has also received some attention. However, research on how ECs integrate multiple biophysical stimuli and the mechanisms underlying this integration remains very limited. In this perspective article, we briefly review what is known about EC responses to multiple synergistic or antagonistic stimuli, and we subsequently present a framework for how an EC may integrate and interpret two physical stimuli to which it is simultaneously subjected. Such a framework promises to advance our understanding of EC integration of multiple biophysical cues which is essential for elucidating the role of endothelial mechanobiology in regulating vascular health and disease.

## 1 Introduction: vascular endothelial mechanobiology

Vascular endothelial cells (ECs), which line the inner surfaces of blood vessels, are continuously subjected to a complex and highly dynamic combination of biophysical cues. Far from being a passive barrier, the endothelium acts as a natural mechanotransducer that constantly senses and responds to its ever-changing mechanical environment [1]. Biophysical stimuli regulate EC organization, structure, and function, playing a pivotal role in maintaining vascular homeostasis. However, when the balance of these mechanical signals is disrupted, the endothelium becomes a central player in the pathogenesis and progression of cardiovascular and metabolic diseases such as atherosclerosis and diabetes [2–4].

The mechanical stress environment to which the endothelium is subjected is complex. Stresses derived from the flowing blood include fluid shear (or frictional) stress, intraluminal pressure, and tensile (stretch) stresses in both the circumferential and longitudinal directions [5, 6]. In addition to these flow-derived cues, ECs are also subjected to substrate-derived contact stresses: the endothelium is anchored to the vascular basement membrane (BM), a specialized extracellular matrix whose multi-scale topography, anisotropic stiffness, and complex curvature modulate EC behavior [5–7].

To date, most *in vitro* studies of EC mechanobiology have focused on responses to single mechanical cues. However, ECs *in vivo* are simultaneously subjected to multiple cues; therefore, understanding the role of EC mechanobiology in regulating vascular physiology and pathology requires elucidating how ECs either synergistically or antagonistically integrate and decipher combinations of biophysical stimuli. In this perspective article, we start by highlighting the difficulty in defining the complex mechanical environment of ECs *in vivo* before briefly reviewing what is known about endothelial responses to combined stimuli and describing major challenges in this field. We conclude with the notion that an EC subjected simultaneously to multiple biophysical cues of a different nature would need to translate these cues into a “common language” that provides a measure of their relative potency and hence dictates how the cell ultimately integrates and interprets a multi-stimulus environment.

## 2 Complexity of the EC mechanical environment

An important feature of the mechanical cues to which ECs are subjected is that they exhibit wide variations in both space and time. For instance, blood pressure, which exhibits significant oscillations between systole and diastole, is on average ∼100 mm Hg (∼13 kPa) in a healthy artery, can rise to ∼200 mm Hg (∼27 kPa) in severe hypertension, and is only ∼10 mm Hg (∼1.3 kPa) in veins [8–12]. Wall shear stress in straight arterial segments exhibits a non-reversing pulsatile waveform with typical time-averaged values of 1 to 2 Pa [13–15], whereas in the vicinity of arterial branches and bifurcations, the flow becomes significantly disturbed, leading to low (0 to 0.5 Pa) shear stress levels and flow directional oscillations [16, 17]. In the presence of severe atherosclerotic disease, wall shear stresses in the stenosed regions can be as high as 10 Pa [18, 19]. Spatial variations associated with geometric cues such as curvature are equally striking: vessel diameters range from 5–10 μm in capillaries to approximately 2.5 cm in the aorta [9, 11]; thus, a ∼30 μm-diameter EC perceives itself as anchored to a flat surface if it is in the aorta but to a highly curved substrate if it is in the microvasculature. These various examples demonstrate that ECs are subjected to vastly different mechanical landscapes depending on their anatomical location, the phase of the cardiac cycle, physical activity level, and disease state.

While certain biophysical stimuli such as blood pressure and wall stretch can be directly measured *in vivo*, accurately measuring wall shear stress *in vivo* remains very difficult due to inaccuracies in near-wall velocity measurement, and assessing vascular wall stiffness and BM topography is equally challenging. Conducting biophysical measurements on excised vessels is also fraught with complications and leads to large variability because the mechanical properties of tissues are intrinsically linked to their biophysical and biochemical environments, which are invariably altered upon excision. For instance, elastic moduli reported for human aortas and coronary arteries span nearly three orders of magnitude, from ∼10 kPa to ∼5 MPa [20–22]. Measurement of BM topography is faced with similar limitations as removal of the endothelium needed to map the BM surface modifies wall curvature and induces swelling and/or retraction, thus altering BM topography in the process [7, 23–26].

Beyond the difficulty of characterizing individual biophysical stimuli, additional challenges arise when considering combinations of cues. Multiple cues are often coupled to one another, rendering it difficult to separate their effects and to identify their relative contributions to the overall EC response. For instance, blood pressure fluctuations during the course of the cardiac cycle also drive temporal fluctuations in wall shear stress as well as cyclic stretching of the vascular wall. When fluid inertial effects are significant, as is the case in medium and large arteries and veins, these three stimuli are phase-shifted relative to one another [5]. Consequently, ECs are continuously subjected to waves of time-varying signals with a multitude of phases. How the cells filter and integrate this complex input information ultimately determines the resulting biological response.

The dynamics of endothelial responses, which can greatly vary for different cues, further complicate our understanding of how the cells integrate multiple biophysical signals. For instance, both shear stress and anisotropic substrate topography induce EC alignment, but they do so with vastly different dynamics: ECs align with topographical cues within minutes [27], whereas complete alignment in response to shear stress typically requires many hours [28]. Thus, when ECs are subjected to both topographical cues and shear stress simultaneously, it is likely that the precise response to shear stress depends on how the endothelium was preconditioned by the topography.

## 3 Endothelial responses to combined biophysical cues

Different experimental systems allow the simultaneous application of multiple mechanical stimuli to ECs. Biomimetic vessel-on-chip systems closely mimic the *in vivo* biomechanical environment, but they often involve a large number of interdependent parameters, rendering it difficult to decouple the stimuli from one another and to understand their combined effects [29–35]. Although such systems will undoubtedly continue to constitute an integral part of the future of *in vitro* modeling, simpler setups involving fewer cues and a more controlled environment promise to facilitate the acquisition of a mechanistic understanding of stimulus integration by ECs.

Studying combinations of biophysical stimuli in pairs allows the identification of synergistic or antagonistic effects without excessive experimental complexity. To date, very few reviews have made the topic of EC responses to combinations of stimuli their principal focus. To bridge this gap, we review studies that have examined the effects on vascular ECs of pairs of the following input stimuli: fluid shear stress, hydrostatic pressure, tensile stress, substrate topography (e.g., 3D-patterned substrates, grooves/ridges, gratings, fibers, etc.), substrate adhesion or geometric confinement (2D adhesive patterns or uniformly coated substrates), substrate curvature, and substrate stiffness. Cellular outputs considered in these studies were grouped into the following four categories (see Table 1): (1) cell size, morphology, orientation, and migration; (2) intracellular organization; (3) cell proliferation and monolayer integrity; and (4) other functional responses.

**Table 1:** Studies of EC responses to pairs of biophysical stimuli, classified into the following four categories of investigated cellular outputs: (1) cell morphology (size, shape, elongation, etc.), cell/nucleus orientation, and cell migration (direction, speed, etc.); (2) intracellular organization (cytoskeleton, polarization, focal adhesion, distribution of organelles, etc.); (3) cell proliferation and monolayer integrity; and (4) other functional responses (gene/protein expression, etc.). Figures were partly generated using Servier Medical Art, provided by Servier and licensed under a Creative Commons Attribution 4.0 License.

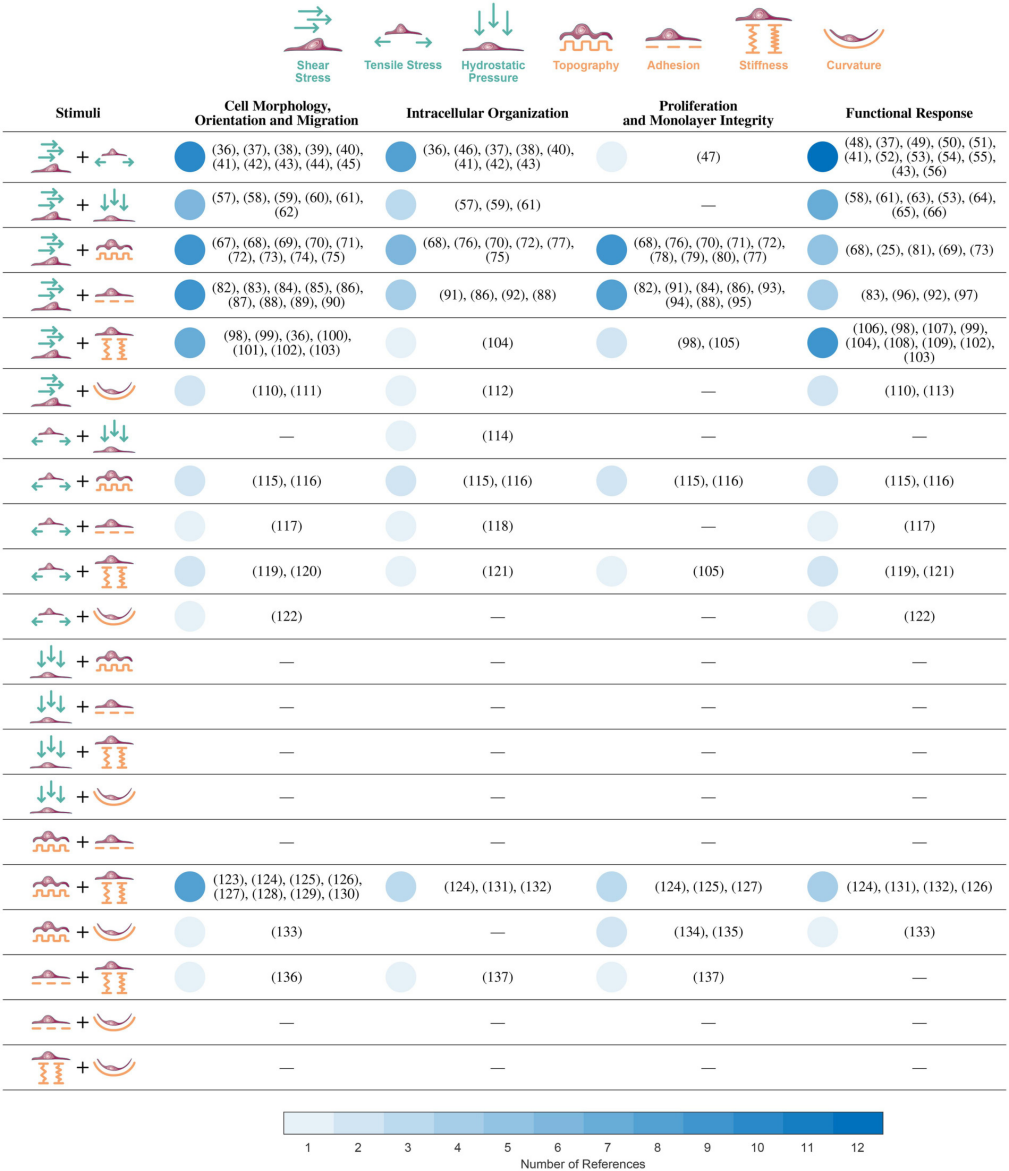

This compilation reveals strong imbalances in the literature (see Table 1). The combination of shear stress with any of the other stimuli with the exception of substrate curvature has been studied the most extensively. Topography has received some attention, particularly combined with shear stress and stiffness, but combinations involving topography with tensile stress or curvature are rare, and combinations with pressure or adhesion have not been explored. Studies combining stiffness with stimuli other than shear stress or topography are also scarce, and other combinations involving tensile stress, confinement, pressure, or curvature remain little studied or virtually unexplored. Importantly, even for the most extensively studied combinations, the number of reports pales in comparison to the extensive literature focusing on the effects of single stimuli.

A similar disparity emerges when considering cellular outputs. Cell morphology, orientation, and migration are by far the most frequently studied responses, likely because they are relatively easy to observe and quantify. Effects on cell function, although arguably more directly relevant physiologically, have been less systematically explored. Depending on the stimulus pair, intracellular organization, monolayer integrity, and cell proliferation have been investigated to varying extents. For example, in response to shear and tensile stresses, intracellular organization has been studied by several groups, while monolayer integrity has received more attention for shear stress combined with adhesion/geometrical confinement. Conversely, there are some combinations, such as shear stress and stretch or stiffness, for which functional responses have been more frequently studied than morphology. It should be noted, however, that even for the combinations that have received the most attention, determining the relative effects of the two stimuli and the mechanisms that underlie EC integration of the dual stimulation remain largely unknown.

Drawing general conclusions from this body of literature remains difficult, mainly because of substantial methodological and experimental differences among the cited studies. Differences in EC type, culture conditions, the manner in which the stimuli are applied, and the cellular responses assessed hinder direct comparisons. For example, substrate topography studies use different materials, are functionalized with different coatings, and have topographic features that vary in shape and dimensions. Similarly, shear stress studies vary in flow type (steady vs. time-dependent, disturbed vs. undisturbed), shear stress magnitude, and flow duration. Combining two stimuli therefore generates a vast experimental parameter space, with very few studies conducted under truly comparable conditions. In addition, the same biological responses are often assessed using different readouts. For instance, a single outcome such as “cell alignment” has at different times been quantified through cell major axis alignment, actin stress fiber alignment, nuclear orientation, cell polarization, or cell migration direction, complicating direct comparisons.

Given the vast number of different stimuli and possible combinations, achieving a comprehensive understanding of endothelial mechanobiology requires a coordinated collective effort across many research groups. This highlights the need for commonly agreeing on model systems and measurement protocols. Wider use of readily available EC types, along with explicit and systematic reporting of key experimental parameters such as cell density, confluence levels, and image acquisition conditions would facilitate comparisons.

## 4 Endothelial integration of multi-stimulus signals

**Figure 1:**
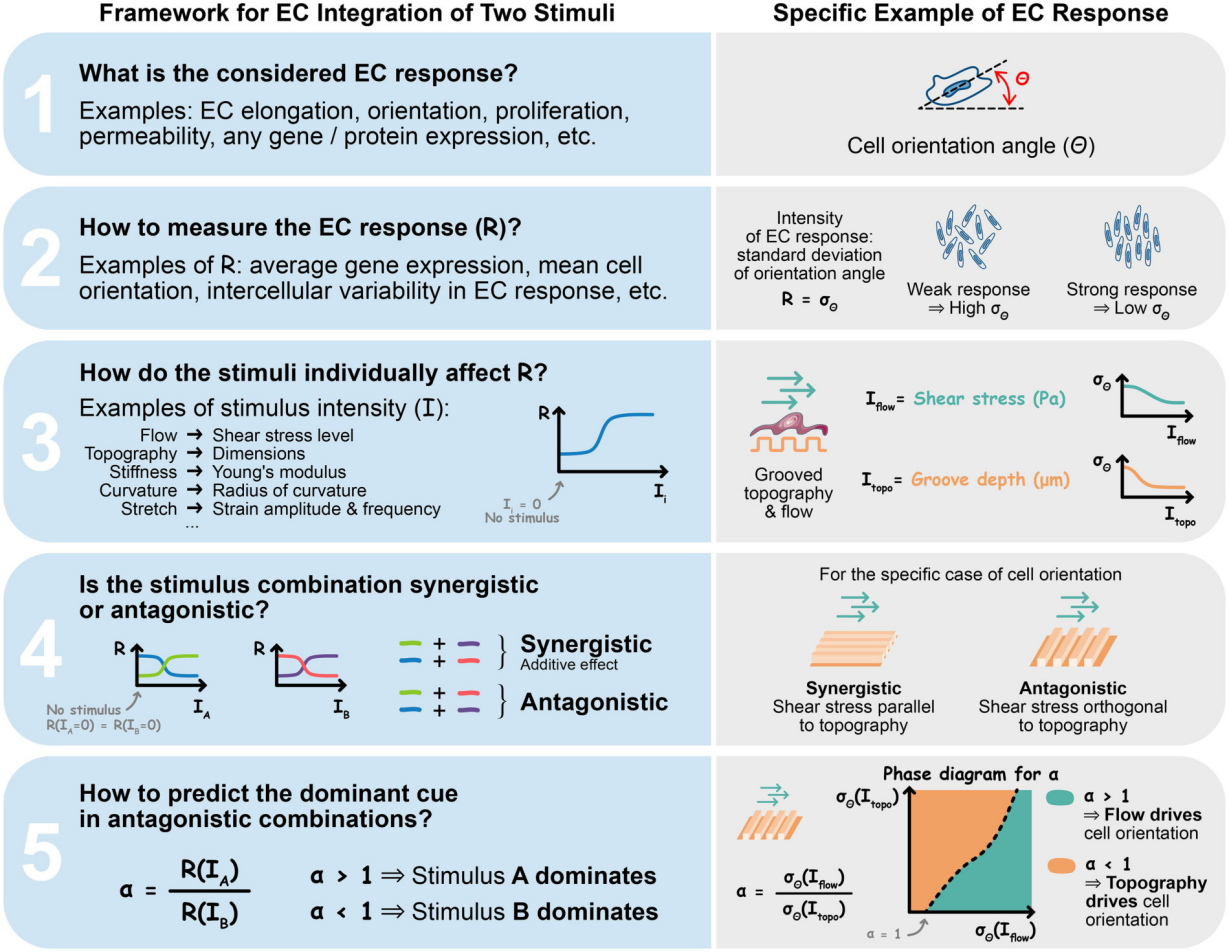
(Left) Framework for EC integration of two antagonistic biophysical stimuli that both individually affect EC response. (Right) Framework applied to the specific example of the EC orientation angle in response to the combination of shear stress and grooved substrate topography.

When subjected simultaneously to multiple biophysical cues, ECs clearly integrate the complex input signal, process the information, and ultimately produce a single adapted output; however, the mechanisms underlying this elaborate signal processing scheme remain to be elucidated. If we consider dual-cue inputs such as the ones outlined in Table 1 where each of the two stimuli individually affects a given cellular output, then the combined impact of the two stimuli may be either synergistic or antagonistic. A synergistic outcome would be expected if the slopes of the considered cellular output with respect to an increase in stimulus intensity have the same sign for both stimuli (see Figure 1). Conversely, slopes of opposite signs would be predicted to lead to an antagonistic outcome. For instance, when ECs are cultured on anisotropic topographic substrates and are simultaneously subjected to shear stress, both of which induce cellular alignment, the two stimuli synergize when the flow is applied in the direction of the topography, potentially amplifying cell alignment, or antagonize when the flow is applied orthogonal to the topography, leading to competition for cell orientation. Interestingly, this same combination of stimuli leads to increased cell elongation regardless of whether the flow is applied parallel or orthogonal to the grooves [74], suggesting that different modes of EC integration of a dual-cue input may exist depending on the specific readout of interest.

Another possible scenario is when only one of the stimuli in a dual-cue input impacts the considered cell response. In that case, the non-triggering stimulus can either potentiate or dampen cell sensitivity to the triggering stimulus, or even completely alter the cell response. For example, cyclic stretch amplifies shear-induced changes in cell area despite having no effect on its own [37]. Hydrostatic pressure alters how ECs orient in response to shear stress, leading to perpendicular rather than parallel alignment at a pressure of 8 kPa [58]. The direction of stretch-induced EC alignment depends on substrate stiffness with cells aligning perpendicular to the strain axis on a substrate with an elastic modulus of 40 kPa but parallel to it on a 200 kPa substrate [119].

## 5 Toward a framework for cell integration of multiple biophysical cues

Integration of multiple biophysical stimuli that can be of a very different nature requires an assessment of the relative intensities of the stimuli. Is there a common “scale” that an EC uses to compare and contrast a shear stress of a certain level with a substrate of a certain stiffness or a particular topographic architecture? Stated alternatively, is there a common “currency” among the different cues that enables cellular integration? If such a currency exists, is it the same for different biological readouts or is it readout-specific?

As a first step towards addressing these highly complex questions, we consider the relatively “simple” case of 2 different biophysical stimuli that have an impact on a given cellular output. If the two stimuli are synergistic, then one would intuitively expect their combined effect to be additive. The more interesting case is when the stimuli are in competition, i.e., when they are expected to have antagonistic effects. In that case, the key question is how to predict which stimulus will dominate the other and hence drive the overall cellular response. If we denote the two stimuli by $S_{\text{A}}$ and $S_{\text{B}}$, their respective intensities by $I_{\text{A}}$ and $I_{\text{B}}$, and the cellular response of interest by R, then one can begin by establishing the dependence of R on $I_{\text{A}}$ and on $I_{\text{B}}$ independently (i.e., $R(I_{\text{A}})$ and $R(I_{\text{B}})$). The ratio $R(I_{\text{A}})/R(I_{\text{B}})$, denoted here as α, would then constitute the most straightforward assessment of the dominance of $S_{\text{A}}$ relative to $S_{\text{B}}$ with α>1 defining the $S_{\text{A}}$-dominated regime, α<1 the $S_{\text{B}}$-dominated regime, and α∼1 the regime where both stimuli have comparable effects on the biological response (see Figure 1). Thus, α can be viewed as some kind of relative potency index that the cell might use to integrate two stimuli of a different nature into a single output. It should be noted that the quantity R here should be interpreted rather broadly; while it can represent the magnitude of the actual response in some cases, it may be better represented by the intercellular variability (variance or standard deviation) of the response in cases where the cell-to-cell variability present in an EC monolayer is expected to decrease as the intensity of the stimulus increases. Naturally, using measures of intercellular variability would be applicable to readouts where this heterogeneity can be measured such as morphometric parameters (cell size, shape, alignment, etc.), immunofluorescence staining of EC monolayers, or spatial transcriptomics. For other readouts such as cell proliferation rate, global gene expression levels, or permeability, an average over the entire population would be a more appropriate measure of the response. An example of this formulation for determining the relative dominance of each of the stimuli in a dual-cue input can be found in the Figure 1.

Naturally, the concept of relative potency index introduced here awaits experimental verification. If validated, then an important question would be how generalizable the concept of relative potency index is in light of the wide spectrum of EC responses and the broad variety of biophysical stimuli that can trigger these responses. Furthermore, we focused here on ECs subjected simultaneously to only two stimuli for the sake of simplicity, but the concept of potency index can, in principle, be extended to any number of stimuli by computing all possible ratios of the readouts in order to establish the dominance or each stimulus relative to the others.

An additional important consideration is the temporal nature of EC mechanobiology. EC responses to mechanical stimulation are dynamic with characteristic time constants that can range from seconds to many hours, depending on the type of stimulus and the nature of the response. For instance, activation of mechanosensitive ion channels occurs within seconds, induction of transcription factors requires minutes, alterations in gene and protein expression typically occur over a few hours, and complete cytoskeletal remodeling requires many hours [138, 139]. This temporal dependence does not fundamentally modify the concept of relative potency index introduced above. In fact, the index itself can be viewed as a dynamic quantity that can be evaluated at any instant in time and can thus provide an appreciation for the relative contributions of individual mechanical stimuli as a function of time.

## 6 Conclusions

The vascular endothelium *in vivo* is constantly subjected to a complex array of mechanical cues. While progress has been made in understanding endothelial responses to individual biophysical stimuli, the integration of multiple, simultaneous cues remains poorly understood. Current research is marked by imbalances and gaps, with shear stress dominating investigations while other stimuli, such as substrate topography, vessel curvature, and hydrostatic pressure, remain understudied. Furthermore, in order to improve comparability across studies, there is a strong need for standardizing experimental models and measurement protocols as well as for systematic reporting of key experimental parameters. To advance our understanding of how ECs subjected simultaneously to multiple biophysical cues interpret this complex input signal, we propose the notion of a relative potency index which provides a quantitative assessment of the relative contributions of the different stimuli to the particular cellular output of interest. This index is an initial step toward establishing a cellular common currency that allows prediction of endothelial responses to multi-cue stimulation. Ultimately, understanding how ECs integrate multiple mechanical signals promises to provide new insight into the central role of EC mechanobiology in vascular health and disease.

## References

[1] Gordon E Schimmel L Frye M. The importance of mechanical forces for in vitro endothelial cell biology. Front Physiol. (2020) 11:684. 10.3389/fphys.2020.00684 32625119 PMC7314997

[2] Hahn C Schwartz MA. Mechanotransduction in vascular physiology and atherogenesis. Nat Rev Mol Cell Biol. (2009) 10:53–62. 10.1038/nrm2596 19197332 PMC2719300

[3] Cheng H Zhong W Wang L Zhang Q Ma X Wang Y, et al. Effects of shear stress on vascular endothelial functions in atherosclerosis and potential therapeutic approaches. Biomed Pharmacother. (2023) 158:114198. 10.1016/j.biopha.2022.114198 36916427

[4] Hamrangsekachaee M Wen K Bencherif SA Ebong EE. Atherosclerosis and endothelial mechanotransduction: current knowledge and models for future research. Am J Physiol Cell Physiol. (2023) 324:C488–C504. 10.1152/ajpcell.00449.2022 36440856 PMC10069965

[5] James BD Allen JB. Vascular endothelial cell behavior in complex mechanical microenvironments. ACS Biomater Sci Eng. (2018) 4:3818–42. 10.1021/acsbiomaterials.8b00628 33429612

[6] Dessalles CA Leclech C Castagnino A Barakat AI. Integration of substrate- and flow-derived stresses in endothelial cell mechanobiology. Commun Biol. (2021) 4:764. 10.1038/s42003-021-02285-w 34155305 PMC8217569

[7] Leclech C Natale CF Barakat AI. The basement membrane as a structured surface – role in vascular health and disease. J Cell Sci. (2020) 133:jcs239889. 10.1242/jcs.239889 32938688

[8] Hall JE Hall ME Guyton AC. Guyton and Hall Textbook of Medical Physiology. 14th ed. Philadelphia, PA: Elsevier (2021).

[9] Gopalan C Kirk E. The blood vessels. In: *Biology of Cardiovascular and Metabolic Diseases*. Elsevier (2022). p. 35–51. 10.1016/B978-0-12-823421-1.00004-4

[10] Whelton PK Carey RM Mancia G Kreutz R Bundy JD Williams B. Harmonization of the American College of Cardiology/American Heart Association and European Society of Cardiology/European Society of hypertension blood pressure/hypertension guidelines: comparisons, reflections, and recommendations. Eur Heart J. (2022) 43:3302–11. 10.1093/eurheartj/ehac432 36100239 PMC9470378

[11] Koeppen BM Stanton BA Hall JM Swiatecka-Urban A, editors. Berne & Levy Physiology. 8th ed. Philadelphia, PA: Elsevier (2024).

[12] McEvoy JW McCarthy CP Bruno RM Brouwers S Canavan MD Ceconi C, et al. 2024 ESC Guidelines for the management of elevated blood pressure and hypertension: developed by the task force on the management of elevated blood pressure and hypertension of the European Society of Cardiology (ESC) and endorsed by the European Society of Endocrinology (ESE) and the European Stroke Organisation (ESO). Eur Heart J. (2024) 45:3912–4018. 10.1093/eurheartj/ehae178 39210715

[13] Moorejr J Xu C Glagov S Zarins C Ku D. Fluid wall shear stress measurements in a model of the human abdominal aorta: oscillatory behavior and relationship to atherosclerosis. Atherosclerosis. (1994) 110:225–40. 10.1016/0021-9150(94)90207-0 7848371

[14] Samijo S Willigers J Barkhuysen R Kitslaar P Reneman R Brands P, et al. Wall shear stress in the human common carotid artery as function of age and gender. Cardiovasc Res. (1998) 39:515–22. 10.1016/S0008-6363(98)00074-1 9798536

[15] Reneman RS Hoeks APG. Wall shear stress as measured in vivo: consequences for the design of the arterial system. Med Biol Eng Comput. (2008) 46:499–507. 10.1007/s11517-008-0330-2 18324431 PMC2441533

[16] Zhao S Ariff B Long Q Hughes A Thom S Stanton A, et al. Inter-individual variations in wall shear stress and mechanical stress distributions at the carotid artery bifurcation of healthy humans. J Biomech. (2002) 35:1367–77. 10.1016/S0021-9290(02)00185-9 12231282

[17] Tarbell JM Shi ZD Dunn J Jo H. Fluid mechanics, arterial disease, and gene expression. Annu Rev Fluid Mech. (2014) 46:591–614. 10.1146/annurev-fluid-010313-141309 25360054 PMC4211638

[18] Gijsen F Katagiri Y Barlis P Bourantas C Collet C Coskun U, et al. Expert recommendations on the assessment of wall shear stress in human coronary arteries: existing methodologies, technical considerations, and clinical applications. Eur Heart J. (2019) 40:3421–33. 10.1093/eurheartj/ehz551 31566246 PMC6823616

[19] Zhang X Jiao Z Hua Z Cao H Liu S Zhang L, et al. Localized elevation of wall shear stress is linked to recent symptoms in patients with carotid stenosis. Cerebrovasc Dis. (2023) 52:283–92. 10.1159/000526872 36273462

[20] Haskett D Johnson G Zhou A Utzinger U Vande Geest J. Microstructural and biomechanical alterations of the human aorta as a function of age and location. Biomech Model Mechanobiol. (2010) 9:725–36. 10.1007/s10237-010-0209-7 20354753

[21] Khanafer K Duprey A Zainal M Schlicht M Williams D Berguer R. Determination of the elastic modulus of ascending thoracic aortic aneurysm at different ranges of pressure using uniaxial tensile testing. J Thorac Cardiovasc Surg. (2011) 142:682–6. 10.1016/j.jtcvs.2010.09.068 21616506

[22] Hooglugt A Klatt O Huveneers S. Vascular stiffening and endothelial dysfunction in atherosclerosis. Curr Opin Lipidol. (2022) 33:353. 10.1097/MOL.0000000000000852 36206080 PMC10128901

[23] Liliensiek SJ Nealey P Murphy CJ. Characterization of endothelial basement membrane nanotopography in rhesus macaque as a guide for vessel tissue engineering. Tissue Eng Part A. (2009) 15:2643–51. 10.1089/ten.tea.2008.0284 19207042 PMC2792116

[24] Peloquin J Huynh J Williams RM Reinhart-King CA. Indentation measurements of the subendothelial matrix in bovine carotid arteries. J Biomech. (2011) 44:815–21. 10.1016/j.jbiomech.2010.12.018 21288524

[25] Jones CG Huang T Chung JH Chen C. 3D-printed, modular, and parallelized microfluidic system with customizable scaffold integration to investigate the roles of basement membrane topography on endothelial cells. ACS Biomater Sci Eng. (2021) 7:1600–7. 10.1021/acsbiomaterials.0c01752 33545000

[26] Barettino A González-Gómez C Gonzalo P Andrés-Manzano MJ Guerrero CR Espinosa FM, et al. Endothelial YAP/TAZ activation promotes atherosclerosis in a mouse model of Hutchinson-Gilford progeria syndrome. J Clin Invest. (2024) 134:e173448. 10.1172/JCI173448 39352768 PMC11563688

[27] Leclech C Krishnamurthy A Muller L Barakat AI. Distinct contact guidance mechanisms in single endothelial cells and in monolayers. Adv Mater Interfaces. (2023) 10:2202421. 10.1002/admi.202202421

[28] Dewey CF Bussolari SR Gimbrone MA Davies PF. The dynamic response of vascular endothelial cells to fluid shear stress. J Biomech Eng. (1981) 103:177–85. 10.1115/1.3138276 7278196

[29] Benbrahim A L’Italien GJ Milinazzo BB Warnock DF Dhara S Gertler JP, et al. A compliant tubular device to study the influences of wall strain and fluid shear stress on cells of the vascular wall. J Vasc Surg. (1994) 20:184–94. 10.1016/0741-5214(94)90005-1 8040941

[30] Tsukurov OI Kwolek CJ L’Italien GJ Benbrahim A Milinazzo BB Conroy NE, et al. The response of adult human saphenous vein endothelial cells to combined pressurized pulsatile flow and cyclic strain, in vitro. Ann Vasc Surg. (2000) 14:260–7. 10.1007/s100169910044 10796958

[31] Casey PJ Dattilo JB Dai G Albert JA Tsukurov OI Orkin RW, et al. The effect of combined arterial hemodynamics on saphenous venous endothelial nitric oxide production. J Vasc Surg. (2001) 33:1199–205. 10.1067/mva.2001.115571 11389418

[32] Estrada R Giridharan GA Nguyen MD Roussel TJ Shakeri M Parichehreh V, et al. Endothelial cell culture model for replication of physiological profiles of pressure, flow, stretch, and shear stress *in vitro*. Anal Chem. (2011) 83:3170–7. 10.1021/ac2002998 21413699

[33] Yu H Kang D Whang M Kim T Kim J. A microfluidic model artery for studying the mechanobiology of endothelial cells. Adv Healthcare Mater. (2021) 10:2100508. 10.1002/adhm.202100508 34297476

[34] Dessalles CA Ramón-Lozano C Babataheri A Barakat AI. Luminal flow actuation generates coupled shear and strain in a microvessel-on-chip. Biofabrication. (2022) 14:015003. 10.1088/1758-5090/ac2baa 34592728

[35] Dessalles CA Cuny N Boutillon A Salipante PF Babataheri A Barakat AI, et al. Interplay of actin nematodynamics and anisotropic tension controls endothelial mechanics. Nat Phys. (2025) 21:999–1008. 10.1038/s41567-025-02847-3 40546251 PMC12176649

[36] Chu PY Hsieh HY Chung PS Wang PW Wu MC Chen YQ, et al. Development of vessel mimicking microfluidic device for studying mechano-response of endothelial cells. iScience. (2023) 26:106927. 10.1016/j.isci.2023.106927 37305698 PMC10251125

[37] Meza D Musmacker B Steadman E Stransky T Rubenstein DA Yin W. Endothelial cell biomechanical responses are dependent on both fluid shear stress and tensile strain. Cell Mol Bioeng. (2019) 12:311–25. 10.1007/s12195-019-00585-0 31719917 PMC6816737

[38] Meza D Abejar L Rubenstein DA Yin W. A shearing-stretching device that can apply physiological fluid shear stress and cyclic stretch concurrently to endothelial cells. J Biomech Eng. (2016) 138:031007. 10.1115/1.4032550 26810848

[39] Sinha R Le Gac S Verdonschot N Koopman B Rouwkema J. Endothelial cell alignment as a result of anisotropic strain and flow induced shear stress combinations. Sci Rep. (2016) 6:29510. 10.1038/srep29510 27404382 PMC4941569

[40] Zheng W Jiang B Wang D Zhang W Wang Z Jiang X. A microfluidic flow-stretch chip for investigating blood vessel biomechanics. Lab Chip. (2012) 12:3441–50. 10.1039/C2LC40173H 22820518

[41] Berardi DE Tarbell JM. Stretch and shear interactions affect intercellular junction protein expression and turnover in endothelial cells. Cell Mol Bioeng. (2009) 2:320–31. 10.1007/s12195-009-0073-7 20161517 PMC2799298

[42] Owatverot TB Oswald SJ Chen Y Wille JJ Yin FCP. Effect of combined cyclic stretch and fluid shear stress on endothelial cell morphological responses. J Biomech Eng. (2005) 127:374–82. 10.1115/1.1894180 16060344

[43] Peng X Recchia FA Byrne BJ Wittstein IS Ziegelstein RC Kass DA. In vitro system to study realistic pulsatile flow and stretch signaling in cultured vascular cells. Am J Physiol Cell Physiol. (2000) 279:C797–C805. 10.1152/ajpcell.2000.279.3.C797 10942730

[44] Zhao S Suciu A Ziegler T Moore JE Bürki E Meister JJ, et al. Synergistic effects of fluid shear stress and cyclic circumferential stretch on vascular endothelial cell morphology and cytoskeleton. Arterioscler Thromb Vasc Biol. (1995) 15:1781–6. 10.1161/01.ATV.15.10.1781 7583556

[45] Moore JE Bürki E Suciu A Zhao S Burnier M Brunner HR, et al. A device for subjecting vascular endothelial cells to both fluid shear stress and circumferential cyclic stretch. Ann Biomed Eng. (1994) 22:416–22. 10.1007/BF02368248 7998687

[46] Shimizu A Goh WH Itai S Hashimoto M Miura S Onoe H. ECM-based microchannel for culturing *in vitro* vascular tissues with simultaneous perfusion and stretch. Lab Chip. (2020) 20:1917–27. 10.1039/D0LC00254B 32307467

[47] Benbrahim A L’Italien GJ Kwolek CJ Petersen MJ Milinazzo B Gertler JP, et al. Characteristics of vascular wall cells subjected to dynamic cyclic strain and fluid shear conditionsin vitro. J Surg Res. (1996) 65:119–27. 10.1006/jsre.1996.0353 8903457

[48] Steadman E Steadman D Rubenstein DA Yin W. Platelet and endothelial cell responses under concurrent shear stress and tensile strain. Microvasc Res. (2024) 151:104613. 10.1016/j.mvr.2023.104613 37793562

[49] Amaya R Pierides A Tarbell JM. The interaction between fluid wall shear stress and solid circumferential strain affects endothelial gene expression. PLoS One. (2015) 10:e0129952. 10.1371/journal.pone.0129952 26147292 PMC4492743

[50] Ando J Yamamoto K. Effects of shear stress and stretch on endothelial function. Antioxid Redox Signal. (2011) 15:1389–403. 10.1089/ars.2010.3361 20854012

[51] Thacher TN Silacci P Stergiopulos N Da Silva RF. Autonomous effects of shear stress and cyclic circumferential stretch regarding endothelial dysfunction and oxidative stress: an ex vivo arterial model. J Vasc Res. (2010) 47:336–45. 10.1159/000265567 20016207

[52] Toda M Yamamoto K Shimizu N Obi S Kumagaya S Igarashi T, et al. Differential gene responses in endothelial cells exposed to a combination of shear stress and cyclic stretch. J Biotechnol. (2008) 133:239–44. 10.1016/j.jbiotec.2007.08.009 17850909

[53] Kwak BR Silacci P Stergiopulos N Hayoz D Meda P. Shear stress and cyclic circumferential stretch, but not pressure, alter connexin43 expression in endothelial cells. Cell Commun Adhes. (2005) 12:261–70. 10.1080/15419060500514119 16531321

[54] Dancu MB Berardi DE Vanden Heuvel JP Tarbell JM. Asynchronous shear stress and circumferential strain reduces endothelial NO synthase and cyclooxygenase-2 but induces endothelin-1 gene expression in endothelial cells. Arterioscler Thromb Vasc Biol. (2004) 24:2088–94. 10.1161/01.ATV.0000143855.85343.0e 15345505

[55] Qiu Y Tarbell JM. Interaction between wall shear stress and circumferential strain affects endothelial cell biochemical production. J Vasc Res. (2000) 37:147–57. 10.1159/000025726 10859473

[56] Ziegler T Silacci P Harrison VJ Hayoz D. Nitric oxide synthase expression in endothelial cells exposed to mechanical forces. Hypertension. (1998) 32:351–5. 10.1161/01.HYP.32.2.351 9719066

[57] Vasanthi Bathrinarayanan P Abadie T Perez Esteban P Vigolo D Simmons MJH Grover LM. Elevated hydrostatic pressure destabilizes VE-cadherin junctions in a time and shear stress dependent manner: an endothelium-on-chip study. APL Bioeng. (2025) 9:036113. 10.1063/5.0275985 40852600 PMC12370293

[58] Mandrycky CJ Ishida T Merkel T Rayner SG Heck A Hadland B, et al. Under pressure: integrated endothelial cell response to hydrostatic and shear stresses. Vasc Biol. (2025) 7:e250015. 10.1530/VB-25-0015 41378902 PMC12741814

[59] Ohashi T Sugaya Y Sakamoto N Sato M. Relative contribution of physiological hydrostatic pressure and fluid shear stress to endothelial monolayer integrity. Biomed Eng Lett. (2016) 6:31–8. 10.1007/s13534-016-0210-x

[60] Liu MC Shih HC Wu JG Weng TW Wu CY Lu JC, et al. Electrofluidic pressure sensor embedded microfluidic device: a study of endothelial cells under hydrostatic pressure and shear stress combinations. Lab Chip. (2013) 13:1743–53. 10.1039/C3LC41414K 23475014

[61] Nakadate H Minamitani H Aomura S. Combinations of hydrostatic pressure and shear stress influence morphology and adhesion molecules in cultured endothelial cells. In: *2010 Annual International Conference of the IEEE Engineering in Medicine and Biology*. Buenos Aires: IEEE (2010). p. 3812–5. 10.1109/IEMBS.2010.5627596 21097057

[62] Sato M Ohashi T. Biorheological views of endothelial cell responses to mechanical stimuli. Biorheology. (2005) 42:421–41. 10.1177/0006355X2005042006003 16369082

[63] Andersson M Karlsson L Svensson PA Ulfhammer E Ekman M Jernås M, et al. Differential global gene expression response patterns of human endothelium exposed to shear stress and intraluminal pressure. J Vasc Res. (2005) 42:441–52. 10.1159/000087983 16155357

[64] Doroudi R Gan LM Selin Sjögren L Jern S. Intraluminal pressure modulates eicosanoid enzyme expression in vascular endothelium of intact human conduit vessels at physiological levels of shear stress. J Hypertens. (2002) 20:63–70. 10.1097/00004872-200201000-00010 11791027

[65] Karmakar N. Interaction of transmural pressure and shear stress in the transport of albumin across the rabbit aortic wall. Atherosclerosis. (2001) 156:321–7. 10.1016/S0021-9150(00)00688-2 11395028

[66] Gan LM Doroudi R Hägg U Johansson AM Selin-Sjögren L Jern S. Differential immediate-early gene responses to shear stress and intraluminal pressure in intact human conduit vessels. FEBS Lett. (2000) 477:89–94. 10.1016/S0014-5793(00)01788-9 10899316

[67] Mancinelli E Taccola S Slay E Chau CCC James N Johnson B, et al. Stable, conductive, adhesive polymer patterning inside a microfluidic chamber for endothelial cell alignment. Adv Mater Technol. (2024) 9:2400404. 10.1002/admt.202400404

[68] Wu X Moimas S Hopf R Giampietro C Kourouklis A Falk V, et al. A free-form patterning method enabling endothelialization under dynamic flow. Biomaterials. (2021) 273:120816. 10.1016/j.biomaterials.2021.120816 33895492

[69] Nakayama KH Surya VN Gole M Walker TW Yang W Lai ES, et al. Nanoscale patterning of extracellular matrix alters endothelial function under shear stress. Nano Lett. (2016) 16:410–9. 10.1021/acs.nanolett.5b04028 26670737 PMC4758680

[70] Whited BM Rylander MN. The influence of electrospun scaffold topography on endothelial cell morphology, alignment, and adhesion in response to fluid flow. Biotechnol Bioeng. (2014) 111:184–95. 10.1002/bit.24995 23842728 PMC3878428

[71] Robotti F Franco D Bänninger L Wyler J Starck CT Falk V, et al. The influence of surface micro-structure on endothelialization under supraphysiological wall shear stress. Biomaterials. (2014) 35:8479–86. 10.1016/j.biomaterials.2014.06.046 25017097

[72] Potthoff E Franco D D’Alessandro V Starck C Falk V Zambelli T, et al. Toward a rational design of surface textures promoting endothelialization. Nano Lett. (2014) 14:1069–79. 10.1021/nl4047398 24428164

[73] Lam RHW Sun Y Chen W Fu J. Elastomeric microposts integrated into microfluidics for flow-mediated endothelial mechanotransduction analysis. Lab Chip. (2012) 12:1865. 10.1039/c2lc21146g 22437210 PMC4120067

[74] Morgan JT Wood JA Shah NM Hughbanks ML Russell P Barakat AI, et al. Integration of basal topographic cues and apical shear stress in vascular endothelial cells. Biomaterials. (2012) 33:4126–35. 10.1016/j.biomaterials.2012.02.047 22417618 PMC3633103

[75] Uttayarat P Chen M Li M Allen FD Composto RJ Lelkes PI. Microtopography and flow modulate the direction of endothelial cell migration. Am J Physiol Heart Circ Physiol. (2008) 294:H1027–35. 10.1152/ajpheart.00816.2007 18156201

[76] Stefopoulos G Robotti F Falk V Poulikakos D Ferrari A. Endothelialization of rationally microtextured surfaces with minimal cell seeding under flow. Small. (2016) 12:4113–26. 10.1002/smll.201503959 27346806

[77] Hwang SY Kwon KW Jang KJ Park MC Lee JS Suh KY. Adhesion assays of endothelial cells on nanopatterned surfaces within a microfluidic channel. Anal Chem. (2010) 82:3016–22. 10.1021/ac100107z 20218573

[78] Hu J Hardy C Chen CM Yang S Voloshin AS Liu Y. Enhanced cell adhesion and alignment on micro-wavy patterned surfaces. PLoS One. (2014) 9:e104502. 10.1371/journal.pone.0104502 25105589 PMC4126693

[79] Franco D Milde F Klingauf M Orsenigo F Dejana E Poulikakos D, et al. Accelerated endothelial wound healing on microstructured substrates under flow. Biomaterials. (2013) 34:1488–97. 10.1016/j.biomaterials.2012.10.007 23182348

[80] Brown A Burke G Meenan BJ. Modeling of shear stress experienced by endothelial cells cultured on microstructured polymer substrates in a parallel plate flow chamber. Biotechnol Bioeng. (2011) 108:1148–58. 10.1002/bit.23022 21125591

[81] Stefopoulos G Giampietro C Falk V Poulikakos D Ferrari A. Facile endothelium protection from TNF-a inflammatory insult with surface topography. Biomaterials. (2017) 138:131–41. 10.1016/j.biomaterials.2017.05.039 28558298

[82] Hoesli CA Tremblay C Juneau PM Boulanger MD Beland AV Ling SD, et al. Dynamics of endothelial cell responses to laminar shear stress on surfaces functionalized with fibronectin-derived peptides. ACS Biomater Sci Eng. (2018) 4:3779–91. 10.1021/acsbiomaterials.8b00774 33429595

[83] Lafaurie-Janvore J Antoine EE Perkins SJ Babataheri A Barakat AI. A simple microfluidic device to study cell-scale endothelial mechanotransduction. Biomed Microdevices. (2016) 18:63. 10.1007/s10544-016-0090-y 27402497

[84] McCracken KE Tran PL You DJ Slepian MJ Yoon JY. Shear- vs. nanotopography-guided control of growth of endothelial cells on RGD-nanoparticle-nanowell arrays. J Biol Eng. (2013) 7:11. 10.1186/1754-1611-7-11 23607894 PMC3637365

[85] Boivin M Chevallier P Hoesli CA Lagueux J Bareille R Rémy M, et al. Human saphenous vein endothelial cell adhesion and expansion on micropatterned polytetrafluoroethylene. J Biomed Mater Res A. (2013) 101A:694–703. 10.1002/jbm.a.34367 22941911

[86] Chollet C Bareille R Rémy M Guignandon A Bordenave L Laroche G, et al. Impact of peptide micropatterning on endothelial cell actin remodeling for cell alignment under shear stress. Macromol Biosci. (2012) 12:1648–59. 10.1002/mabi.201200167 23169680

[87] Lin X Helmke BP. Cell structure controls endothelial cell migration under fluid shear stress. Cell Mol Bioeng. (2009) 2:231–43. 10.1007/s12195-009-0060-z 23181134 PMC3505107

[88] Vartanian KB Kirkpatrick SJ Hanson SR Hinds MT. Endothelial cell cytoskeletal alignment independent of fluid shear stress on micropatterned surfaces. Biochem Biophys Res Commun. (2008) 371:787–92. 10.1016/j.bbrc.2008.04.167 18471992

[89] Lin X Helmke BP. Micropatterned structural control suppresses mechanotaxis of endothelial cells. Biophys J. (2008) 95:3066–78. 10.1529/biophysj.107.127761 18586851 PMC2527245

[90] Hsu S Thakar R Liepmann D Li S. Effects of shear stress on endothelial cell haptotaxis on micropatterned surfaces. Biochem Biophys Res Commun. (2005) 337:401–9. 10.1016/j.bbrc.2005.08.272 16188239

[91] Gong X Yao J He H Zhao X Liu X Zhao F, et al. Combination of flow and micropattern alignment affecting flow-resistant endothelial cell adhesion. J Mech Behav Biomed Mater. (2017) 74:11–20. 10.1016/j.jmbbm.2017.04.028 28525819

[92] Vartanian KB Berny MA McCarty OJT Hanson SR Hinds MT. Cytoskeletal structure regulates endothelial cell immunogenicity independent of fluid shear stress. Am J Physiol Cell Physiol. (2010) 298:C333–41. 10.1152/ajpcell.00340.2009 19923423

[93] Zorlutuna P Rong Z Vadgama P Hasirci V. Influence of nanopatterns on endothelial cell adhesion: enhanced cell retention under shear stress. Acta Biomater. (2009) 5:2451–9. 10.1016/j.actbio.2009.03.027 19394284

[94] Young EWK Wheeler AR Simmons CA. Matrix-dependent adhesion of vascular and valvular endothelial cells in microfluidic channels. Lab Chip. (2007) 7:1759. 10.1039/b712486d 18030398

[95] Feugier P Black R Hunt J How T. Attachment, morphology and adherence of human endothelial cells to vascular prosthesis materials under the action of shear stress. Biomaterials. (2005) 26:1457–66. 10.1016/j.biomaterials.2004.04.050 15522747

[96] Wang C Baker BM Chen CS Schwartz MA. Endothelial cell sensing of flow direction. Arterioscler Thromb Vasc Biol. (2013) 33:2130–6. 10.1161/ATVBAHA.113.301826 23814115 PMC3812824

[97] Wu CC Li YS Haga JH Kaunas R Chiu JJ Su FC, et al. Directional shear flow and Rho activation prevent the endothelial cell apoptosis induced by micropatterned anisotropic geometry. Proc Natl Acad Sci. (2007) 104:1254–9. 10.1073/pnas.0609806104 17229844 PMC1783086

[98] Yates AK Murray H Kjar A Chavarria D Masters H Kim H, et al. Substrate stiffness and shear stress collectively regulate the inflammatory phenotype in cultured human brain microvascular endothelial cells. Fluids Barriers CNS. (2025) 22:73. 10.1186/s12987-025-00683-4 40665329 PMC12261575

[99] Lai A Zhou Y Thurgood P Chheang C Chandra Sekar N Nguyen N, et al. Endothelial response to the combined biomechanics of vessel stiffness and shear stress is regulated via piezo1. ACS Appl Mater Interfaces. (2023) 15:59103–16. 10.1021/acsami.3c07756 38073418

[100] James BD Allen JB. Sex-specific response to combinations of shear stress and substrate stiffness by endothelial cells in vitro. Adv Healthc Mater. (2021) 10:2100735. 10.1002/adhm.202100735 PMC8458248 34142471

[101] Bacci C Wong V Barahona V Merna N. Cardiac and lung endothelial cells in response to fluid shear stress on physiological matrix stiffness and composition. Microcirculation. (2021) 28:e12659. 10.1111/micc.12659 32945052

[102] Kohn JC Zhou DW Bordeleau F Zhou AL Mason BN Mitchell MJ, et al. Cooperative effects of matrix stiffness and fluid shear stress on endothelial cell behavior. Biophys J. (2015) 108:471–8. 10.1016/j.bpj.2014.12.023 25650915 PMC4317546

[103] Galie PA Van Oosten A Chen CS Janmey PA. Application of multiple levels of fluid shear stress to endothelial cells plated on polyacrylamide gels. Lab Chip. (2015) 15:1205–12. 10.1039/C4LC01236D 25573790 PMC4500630

[104] Walther BK Rajeeva Pandian NK Gold KA Kiliç ES Sama V Gu J, et al. Mechanotransduction-on-chip: vessel-chip model of endothelial YAP mechanobiology reveals matrix stiffness impedes shear response. Lab Chip. (2021) 21:1738–51. 10.1039/D0LC01283A 33949409 PMC9761985

[105] Omid H Abdollahi S Bonakdar S Haghighipour N Shokrgozar MA Mohammadi J. Biomimetic vascular tissue engineering by decellularized scaffold and concurrent cyclic tensile and shear stresses. J Mater Sci Mater Med. (2023) 34:12. 10.1007/s10856-023-06716-4 36917304 PMC10014704

[106] Lai A Zhou Y Chheang C Mirabedini A Mirzaalikhan Y Noonan J, et al. Decoding vascular aging: Substrate stiffness and shear stress orchestrate endothelial inflammation and remodelling via mechanosensitive pathways. Biomaterials. (2026) 329:123932. 10.1016/j.biomaterials.2025.123932 41435451

[107] Hamrangsekachaee M Chen Y Tressler ER McCauley L O’Hare NR Okorafor CC, et al. Engineering mechanical microenvironments: integration of substrate and flow mechanics reveals the impact on the endothelial glycocalyx. ACS Biomater Sci Eng. (2025) 11:3416–31. 10.1021/acsbiomaterials.4c02401 40434411 PMC12152830

[108] Li W Li P Li N Du Y Lü S Elad D, et al. Matrix stiffness and shear stresses modulate hepatocyte functions in a fibrotic liver sinusoidal model. Am J Physiol Gastrointest Liver Physiol. (2021) 320:G272–82. 10.1152/ajpgi.00379.2019 33296275 PMC8609567

[109] Mina SG Huang P Murray BT Mahler GJ. The role of shear stress and altered tissue properties on endothelial to mesenchymal transformation and tumor-endothelial cell interaction. Biomicrofluidics. (2017) 11:044104. 10.1063/1.4991738 28798857 PMC5533495

[110] Mandrycky CJ Abel AN Levy S Marsh LM Chassagne F Chivukula VK, et al. Endothelial responses to curvature-induced flow patterns in engineered cerebral aneurysms. J Biomech Eng. (2023) 145:011001. 10.1115/1.4054981 35838329 PMC9445320

[111] Ye M Sanchez HM Hultz M Yang Z Bogorad M Wong AD, et al. Brain microvascular endothelial cells resist elongation due to curvature and shear stress. Sci Rep. (2014) 4:4681. 10.1038/srep04681 24732421 PMC3986701

[112] Frame MD Sarelius IH. Flow-induced cytoskeletal changes in endothelial cells growing on curved surfaces. Microcirculation. (2000) 7:419–27. 10.1111/j.1549-8719.2000.tb00140.x 11142339

[113] Van Epps JS Chew DW Vorp DA. Effects of cyclic flexure on endothelial permeability and apoptosis in arterial segments perfused ex vivo. J Biomech Eng. (2009) 131:101005. 10.1115/1.3192143 19831475

[114] Newton JD Hossain S Abedi K Song Y Pavelick J. Tunable stiffness of matrix-derived membranes enables independent and coupled analysis of pressure and strain effects in barrier tissue-on-chip models. ACS Appl Mater Interfaces. (2025) 17:67543–58. 10.1021/acsami.5c15966 41348548

[115] Shi Y Li D Yi B Tang H Xu T Zhang Y. Physiological cyclic stretching potentiates the cell–cell junctions in vascular endothelial layer formed on aligned fiber substrate. Biomater Adv. (2024) 157:213751. 10.1016/j.bioadv.2023.213751 38219418

[116] Ren C Chang Z Li K Wang X Wang D Xu Y, et al. Impact of uniaxial cyclic stretching on matrix-associated endothelial cell responses. Mater Today Bio. (2024) 27:101152. 10.1016/j.mtbio.2024.101152 39104901 PMC11298614

[117] Balachandran K Alford PW Wylie-Sears J Goss JA Grosberg A Bischoff J, et al. Cyclic strain induces dual-mode endothelial-mesenchymal transformation of the cardiac valve. Proc Natl Acad Sci. (2011) 108:19943–8. 10.1073/pnas.1106954108 22123981 PMC3250145

[118] Katanosaka Y Bao JH Komatsu T Suemori T Yamada A Mohri S, et al. Analysis of cyclic-stretching responses using cell-adhesion-patterned cells. J Biotechnol. (2008) 133:82–9. 10.1016/j.jbiotec.2007.09.017 17981352

[119] Chandra Sekar N Aguilera Suarez S Nguyen N Lai A Thurgood P Zhou Y, et al. Studying the synergistic effect of substrate stiffness and cyclic stretch level on endothelial cells using an elastomeric cell culture chamber. ACS Appl Mater Interfaces. (2023) 15:4863–72. 10.1021/acsami.2c15818 36652631

[120] Korff T Augustin HG. Tensional forces in fibrillar extracellular matrices control directional capillary sprouting. J Cell Sci. (1999) 112:3249–58. 10.1242/jcs.112.19.3249 10504330

[121] Dan A Huang RB Leckband DE. Dynamic imaging reveals coordinate effects of cyclic stretch and substrate stiffness on endothelial integrity. Ann Biomed Eng. (2016) 44:3655–67. 10.1007/s10439-016-1677-4 27317301 PMC5114158

[122] Zeinali S Thompson EK Gerhardt H Geiser T Guenat OT. Remodeling of an *in vitro* microvessel exposed to cyclic mechanical stretch. APL Bioeng. (2021) 5:026102. 10.1063/5.0010159 33834157 PMC8019357

[123] Islam T Hooper J Zhang X Garcia C Hasan MM Drewry DH, et al. A soft-stiff patterned bioengineering model reveals kinase pathways driving directional cell migration in pulmonary arterial hypertension. Biomater Sci. (2025) 13:5671–84. 10.1039/D5BM00224A 40904251 PMC12409394

[124] Yi B Shen Y Tang H Wang X Zhang Y. Stiffness of the aligned fibers affects structural and functional integrity of the oriented endothelial cells. Acta Biomater. (2020) 108:237–49. 10.1016/j.actbio.2020.03.022 32205213

[125] Merkle VM Tran PL Hutchinson M Ammann KR DeCook K Wu X, et al. Core–shell PVA/gelatin electrospun nanofibers promote human umbilical vein endothelial cell and smooth muscle cell proliferation and migration. Acta Biomater. (2015) 27:77–87. 10.1016/j.actbio.2015.08.044 26320540

[126] Jeon H Tsui JH Jang SI Lee JH Park S Mun K, et al. Combined effects of substrate topography and stiffness on endothelial cytokine and chemokine secretion. ACS Appl Mater Interfaces. (2015) 7:4525–32. 10.1021/acsami.5b00554 25658848 PMC4937831

[127] Kim P Yuan A Nam KH Jiao A Kim DH. Fabrication of poly(ethylene glycol): gelatin methacrylate composite nanostructures with tunable stiffness and degradation for vascular tissue engineering. Biofabrication. (2014) 6:024112. 10.1088/1758-5082/6/2/024112 24717683

[128] Dickinson LE Rand DR Tsao J Eberle W Gerecht S. Endothelial cell responses to micropillar substrates of varying dimensions and stiffness. J Biomed Mater Res A. (2012) 100A:1457–66. 10.1002/jbm.a.34059 PMC3324647 22389314

[129] Charest JM Califano JP Carey SP Reinhart-King CA. Fabrication of substrates with defined mechanical properties and topographical features for the study of cell migration. Macromol Biosci. (2012) 12:12–20. 10.1002/mabi.201100264 22021131

[130] Feinberg AW Wilkerson WR Seegert CA Gibson AL Hoipkemeier-Wilson L Brennan AB. Systematic variation of microtopography, surface chemistry and elastic modulus and the state dependent effect on endothelial cell alignment. J Biomed Mater Res A. (2008) 86A:522–34. 10.1002/jbm.a.31626 17994556

[131] VanderBurgh JA Potharazu AV Schwager SC Reinhart-King CA. A discrete interface in matrix stiffness creates an oscillatory pattern of endothelial monolayer disruption. J Cell Sci. (2020) 133:244533. 10.1242/jcs.244533 PMC7520461 32878941

[132] VanderBurgh JA Hotchkiss H Potharazu A Taufalele PV Reinhart-King CA. Substrate stiffness heterogeneities disrupt endothelial barrier integrity in a micropillar model of heterogeneous vascular stiffening. Integr Biol. (2018) 10:734–46. 10.1039/C8IB00124C PMC6301132 30382278

[133] Fu R Jones E Chen N Sun B Si B Wei ZA, et al. Multiscale 3D printing of nanoporous scaffolds with surface topography for guiding 3D cell alignment. Adv Healthc Mater. (2025) 15:e04630. 10.1002/adhm.202504630 41117090

[134] Wang Y Chen S Pan Y Gao J Tang D Kong D, et al. Rapid in situ endothelialization of a small diameter vascular graft with catalytic nitric oxide generation and promoted endothelial cell adhesion. J Mater Chem B. (2015) 3:9212–22. 10.1039/C5TB02080H 32263136

[135] Uttayarat P Perets A Li M Pimton P Stachelek SJ Alferiev I, et al. Micropatterning of three-dimensional electrospun polyurethane vascular grafts. Acta Biomater. (2010) 6:4229–37. 10.1016/j.actbio.2010.06.008 20601235

[136] Versaevel M Riaz M Corne T Grevesse T Lantoine J Mohammed D, et al. Probing cytoskeletal pre-stress and nuclear mechanics in endothelial cells with spatiotemporally controlled (de-)adhesion kinetics on micropatterned substrates. Cell Adhes Migr. (2017) 11:98–109. 10.1080/19336918.2016.1182290 PMC5308222 27111836

[137] Feinberg AW Schumacher JF Brennan AB. Engineering high-density endothelial cell monolayers on soft substrates. Acta Biomater. (2009) 5:2013–24. 10.1016/j.actbio.2009.01.032 19269269

[138] Davies PF. Flow-mediated endothelial mechanotransduction. Physiol Rev. (1995) 75:519–60. 10.1152/physrev.1995.75.3.519 7624393 PMC3053532

[139] Barakat AI. Blood flow and arterial endothelial dysfunction: mechanisms and implications. C R Phys. (2013) 14:479–96. 10.1016/j.crhy.2013.05.003

